# Report of a Lecture on Dysentery, Delivered by Dr. Gerhard, at the Philadelphia Hospital, Blockley, November 16th

**Published:** 1839-11-23

**Authors:** 


### CLINICAL LECTURE.

### REPORT OF A LECTURE ON DYSENTERY, *delivered by* Dr. Gerhard, *at the Philadelphia Hospital, Blockley, November 16th.*

Dysentery is a disease of unfrequent occurrence in the cold seasons of the year. It is most commonly met with in summer and fall, the liability of inflammation being transferred with the approach to winter, from the bowels to the lungs. Dr. Gerhard, however, presented two cases of dysentery, one of the acute, the other of the subacute form.

The latter was that of a man aged sixty-five years; he has generally enjoyed good health: on his first admission into the hospital, he had intermittent fever, from which he recovered, and went about his usual employment. About a fortnight after this, (on the 1st of October,) he was again admitted, having been seized with dysentery two days before. It came on with frequent discharges from the bowels, which were watery, and passed with little pain. In a few days the character of the stools changed; they became yellowish, and were composed of thin fœcal matter, mixed with mucus; but there was *no blood.* The patient has also suffered pains, but of no great severity, along the course of the colon, from the cœcum to the sigmoid flexure. He has not experienced nausea; his appetite has been tolerable; he has suffered little from thirst. The skin has been harsh and dry, with considerable emaciation, and a countenance indicative of griping pain in the bowels; the features which give to it this expression, are the frown on the brow and compression of the lips. The degree of emaciation has varied frequently with the intensity of the case; being on one day extreme,—the next much diminished. The pulse has been sometimes quick, sometimes low; it now beats 96 in the minute. The skin is cool; there is, therefore, very little fever, nor has there been much at any time in the course of the disease. The tongue has been, throughout, dry, cracked, and red, as it almost always is, in severe cases of *chronic* dysentery. This appearance of the tongue is not so frequent in *acute* dysentery, because the inflammation requires some time to extend itself up the alimentary canal. In chronic cases we often find this condition of the tongue attended with a disagreeable taste, and even ulcers in the mouth. The patient's tongue is now become natural; abdomen slightly tender, and not retracted.

The *acute* case is that of a woman forty years of age. She was admitted on the 12th of November. During the summer she had an attack of dysentery, and has since been confined in the wards with rheumatism, but had recovered. Her present illness commenced on the 10th inst. The discharges were frequent and watery; on the 12th they contained mucus with some blood. She has had fever, but no chills; nausea, but no vomiting.

15*th* —The countenance is anxious; abdomen extremely tender and painful; stools passed every hour; they contain mucus, but no blood.

To-day (16th) the blood has reappeared in the stools. This disappearance and reappearance of blood in the stools, are of frequent occurrence in acute dysentery. The history of the case shows that the stomach has remained nearly intact, the disease being confined to the large intestine.

*Present condition of the patient.*—The countenance is very slightly flushed, especially the lips; there is no compression of the lips, as in the former case; the countenance expresses nausea and disgust, rather than griping pain. The skin is moist and pleasant, but has been warm and less moist. The tongue is covered with a brownish fur, but moist; there is pain, on pressure, all over the abdomen, but it is especially severe in the transverse colon, and sigmoid flexure; pulse moderately strong, but compressible, and beats 110. The intellect is confused and weak, but this condition is habitual to this woman, and is not connected with the disease.

Dr. G. then presented a case of tubercular diarrhœa; a disease having a close analogy to dysentery. The disease has continued for two months, the patient having for some time previous been labouring under phthisis pulmonalis. Since the commencement of the diarrhœa the pain in the chest has continued, but the cough has declined, as mostly occurs in such cases. The diarrhœa seems to act uniformly as a revulsive, and stills the cough, or sometimes removes it for a time. The patient passes five or six stools daily; they consist of ordinary fœcal matter, mixed with serum, but *no mucus or blood.*

The *diagnosis* of dysentery, is in general, easy in acute cases. The tormina and tenesmus, and peculiar stools are sufficient to distinguish it. But in the chronic form of the disease, the diagnosis is more difficult, as it is apt to be confounded with that form of diarrhœa which is produced by a tubercular condition of the follicles of the small and large intestine, and is usually preceded by a similar condition of the lungs. We are to distinguish them by the history of the case. Tubercular diarrhœa is, in most cases preceded by phthisis pulmonalis, that is, the disease generally begins in the lungs before it attacks the bowels. The discharges are generally irregular as to amount and frequency, and they differ in nature also from those of dysentery, as is proved by reference to the above cases.

*Anatomical lesions.*—Dysentery is an inflammation of the *large intestine*, as is sufficiently indicated by the position of the pain. This inflammation and its consequences in some cases extend a short distance into the small intestine, and even to the stomach; but it always commences in the large intestine, and is generally confined to it. It mostly begins towards the lower end of the colon, and is sometimes restricted altogether to within a short distance from the anus. The inflammation produces ulceration in various degrees; thickening of the mucous, and other coats; contraction of the calibre of the intestine, from the spasm of the muscular fibres, or sloughing of the mucous membrane, which may thus be extensively detached. The mucous follicles suffer much from the disease, and the ulceration generally begins in them, and then assumes a regularly rounded form; then smaller ulcers run together, and finally give rise to the extensive destruction of the mucous coat which occurs in most bad cases of dysentery. The anatomical lesions of this disease are of importance for the prognosis; for when you have become familiar with them you may readily understand how slow the intestine is to recover its normal condition; indeed, it is apt to remain for a long time more or less diseased, notwithstanding the diminution of the symptoms. The contraction of the gut is one of the greatest obstacles to perfect cure when the ulceration has been extensive, for it can no longer bear the distention caused by the passage of fæcal matter, and every new process of defecation is a new irritant to the denuded surface. It is, however, surprising to find that the intestine will sometimes, though rarely, regain a healthy state after the most extensive sloughing and ulceration. That is, it will regain very nearly a normal condition, but, perhaps, remain a little more irritable than usual. These remarks are applicable to the protracted cases of the disease, where the ulceration is deeply seated, and the powers of restoration have declined. When the disease is acute, the most extended ulcers will cicatrize kindly, and leave behind a smooth cicatrix, with puckered edges. These I have often seen months and years after an attack of acute dysentery, in patients who have died of diseases in no way connected with it. The depth of the ulcers is, therefore, more important than their extent.

*Treatment of dysentery.*—In the acute form of the disease, the treatment is sufficiently simple. The usual antiphlogistic means are required, with local applications to the inflamed mucous membrane, calculated to allay its irritability and remove its morbid secretions: these local remedies are narcotics and laxatives. In the practice of this hospital, especially during the present year, we rarely find it necessary to bleed. We give first a dose of castor oil, and then make use of the oily mixture. Calomel, either alone, or combined with opium or ipecacuanha, is by far the best remedy in severe cases; we sometimes also use ipecacuanha alone, or Dover's powder. In most cases mercurials are sufficient to effect a cure as soon as they produce ptyalism, when the symptoms of acute dysentery often cease at once. Half or a quarter of a grain of calomel, every two hours, will salivate in three or four days. It is usually combined with opium, to allay the griping, and prevent purging; or the pulv. ipecac. et opii may be employed in place of the opium, to effect the same objects. We have also frequently used ipecacuanha, either alone, or combined with opium or calomel. In the case of subacute dysentery before us, we have employed these articles, at times resorting to the acetate of lead, and various astringents, without much advantage: Dover's powder has produced the most benefit. In the acute case, we have administered half a grain of calomel, with three grains of Dover's powder, every two hours.

We rarely employ calomel as a *purgative* in this disease. We use it for a few days only, to produce its specific antiphlogistic effect,—that is, until slight ptyalism is induced. If it is not then attended with good effects, it should be given up: a continuance of its use will do much injury, and tend to increase the ulceration of the bowels.

This is a peculiarity in the action of mercurials; in many acute inflammatory diseases, the advantages to be gained are when the point of salivation is reached, which is a test of the operation of the disease, and the system may then be regarded as saturated. I am quite convinced that if, from any peculiarity of the system, or from the disease assuming an unusual tendency to the spreading of the ulcerations, mercury should be administered after ptyalism has been produced without benefit, the patient is decidedly injured. The remedy is best adapted to the inflammatory forms of the disorder, and, as we shall presently see, is least fitted for the sloughing or malignant variety.

Of the particular remedies in dysentery, purgatives have been extensively employed. We have used many articles of this class; the best we find to be castor oil, which purges sufficiently to carry off the vitiated secretions, without producing much irritation. To prevent the oil from acting too harshly, and to lessen the irritability of the bowels, laudanum may be advantageously combined with it. The oleaginous mixture is a good formula for their combination; of this we may give half an ounce every two hours, till it begins to act on the bowels. Rhubarb will also answer well as a purgative, and when the active symptoms have declined, the spiced syrup answers better than any other remedy. Venesection is sometimes required in acute dysentery, when the pulse is strong and corded; but we have not found it necessary in any case which has occurred in this hospital during the present year. The epidemic character of the disease has not been of the violent inflammatory character, which is a cardinal point in the diseases of the mucous surfaces, and seems necessary to the perfect cessation of the disease. I would not have you to misunderstand me, the term, restoration of the secretions, has been much abused and used vaguely. It means simply, in this case, to bring about the natural secretions of mucus, &c., in place of the diseased ones of blood and lymph. A certain set of remedies tend directly to produce this effect, and by restoring the natural secretions, they not only prove that the disease is ceasing, but they contribute to its cessation by producing depletion in the most effectual way, that is, through the natural emunctories of the part. Cups and leeches to the abdomen, along the course of the colon, are also frequently advisable; the latter may also be applied around the anus, for the purpose of drawing blood from the hæmorrhoidal vessels, and relieving the tenesmus. Warm fomentations are very often beneficially employed. But these measures, however important, cannot alone be relied on for the cure of the disease; we must restore the secretions to their healthy condition. This is a principal, though not the only object for which we employ calomel, with opiates, &c. The action of opium in dysentery is peculiar; in the first place, it allays the local pain and general irritabillity; and secondly, it quiets the spasmodic movements of the intestine, and thereby facilitates the process of cicatrization. But it may likewise produce bad effects; it tends to lock up the bowels, and prevent the discharge of the morbid secretions. To obviate this disadvantage we seldom use it alone, but combine it with castor oil, calomel, or ipecac. It may sometimes, however, be employed singly, either at the commencement or towards the close of the disease; but never during the height of the inflammation. Opium is also used by *injection.* In this city, opiate injections in dysentery have not been much employed till within the last few years; and in the country their use is still very limited, but in this hospital we are in the habit of using them very largely. From twenty to forty drops of laudanum may be administered in this way, but not more, for dangerous consequences from time to time result from the frequent employment of large quantities of so powerful a narcotic, particularly when given by the rectum, in which mode of administration its action upon the brain is more irregular than when given in any other way. We usually inject twenty drops of laudanum mixed with a small portion of mucilage, every two, three, or four hours, according to the severity of the tenesmus, and the effects of the remedy; thus, if the stools cease, or if the mind becomes confused, dull, or the patient sleepy, its use should be suspended. There is still another way in which opium may be employed in dysentery; that is, by means of poultices sprinkled with laudanum, and applied to the abdomen.

Of the other remedies employed in dysentery, ipecacuanha, as we have already mentioned, is among the most useful. It is used either singly or combined with calomel or opium. A very effectual method of administering it, is in combination with extract of gentian and blue mass. This combination originated with Mr. Twining, and has been extensively and beneficially employed in India. It generally produces vomiting at first, but in a short time this effect ceases. We have tried it in one epidemic; its administration was followed by nausea and diaphoresis, and a considerable alleviation of the symptoms. It sometimes failed, but was generally successful. The proportions are, six grains of ipecacuanha, four of blue mass, and five of the extract of gentian.

Various other remedies have been employed in acute dysentery. They are principally depletions, such as saline purgatives, calomel in large doses, &c. These will doubtless answer in many of the ordinary cases of the disease.

*Malignant* Dysentery is a form of the disease requiring considerable modification in the treatment. It occurs for the most part in hospitals, ships, camps, &c. We had an epidemic of it in Philadelphia in 1837, and some cases in 1838. It is so violent and rapid in its progress as sometimes to produce gangrene of the intestine in two days. It is attended with great prostration of the vital powers; subsultus tendinum, and various other signs of debility and nervous disorder. All modes of treatment will frequently fail in this form of the disease. In the epidemic of 1837, we found it necessary to resort to stimuli, tonics and astringents; as wine or brandy, cinchona or cascarilla, with the early use of kino, catechu or chalk. Opium was also employed as a stimulus.

Another variety is the *subacute*, of which we have an example in the first case above detailed. It occurs mostly in persons above the age of forty; and appears to be the effect of irregular habits, or of the gradual decline of the powers of life. In these cases, besides a regulated diet, we find Dover's powder to be the most effectual remedy; it succeeds better than mercurials. We have given it in three grain doses every four or six hours. Cases of subacute dysentery are unfrequent in summer, being most commonly met with in the fall. We have had many cases of it in this hospital; they have been principally confined to the lunatic wards—a circumstance which is explained by the debilitating effect which a disordered mind has upon the system.

Besides the remedies already spoken of, the *acids* have been largely used in the treatment of dysentery. This practice originated in tropical climates, where lime juice, vinegar, and other vegetable acids were employed. The use of the mineral acids was introduced by Dr. Hope, whose mixture of nitrous acid, camphor and laudanum, has been of late years so extensively used in bowel diseases. It often produces the best effects, but will not answer in the sloughing form of the disease. It proves most effectual in the subacute variety, and sometimes in the acute, after the severity of the case has declined; but in the ordinary cases of acute dysentery, the benefit produced by this mixture is very problematical. The dose is about half an ounce every two or three hours.

The acid practice is founded upon a peculiar change in the symptoms of the disease which occurs in dysentery. The stools and saliva become extremely alkaline, and even the urine and perspiration lose to a certain extent or altogether their excess of acid. In giving the mixture I have usually continued its administration until the excessive alkalinity of the secretions had diminished or altogether ceased.

*Chronic* Dysentery is another form which we frequently meet with. We have a case of it at present, in a woman who has been suffering with it for six or seven weeks. There has been griping in the region of the transverse colon, but during the last week it has been slight: there have been three discharges in the last ten or twelve hours; the skin is dry and harsh; the patient is much emaciated; this form of the affection, indeed produces more emaciation than any other disease except cancer. Chronic dysentery may last for years, and produce extensive ulceration or sloughing; and when once checked, is very liable to return.

*Treatment of Chronic Dysentery.*—We must rely principally upon a regulated *diet*, of such a nature as will best agree with the patient; in most cases farinaceous articles answer best, while others require animal food. Of the remedies to be employed, the best are opium and ipecacuanha; calomel, in minute doses, will also prove useful. In many cases, travelling by land or sea, particularly the latter, has operated very beneficially, by producing a general alteration and improvement in the system. This has been found to be particularly the case in the dysentery of the East Indies.

From the preceding remarks you will understand that our treatment of dysentery must vary exceedingly in the different forms of the disease. The success of the treatment will, therefore, be various in different epidemics. In the malignant, sloughing dysentery which occurs in camps, &c., the mortality is generally great, while, in some epidemics, it is comparatively trifling; we should not form a general opinion of the character of the disease from observation of a single epidemic, and still less, can we estimate the success of our treatment, unless it has been tested in various epidemics and in different years. A multitude of remedies are often prescribed and used with great benefit in the treatment of the disease; the limits of this lecture will prevent me from even mentioning the greater part of them, but they will be in general suggested by the peculiar symptoms of each case, and you will often succeed in the most obstinate cases, by attending to some apparently unimportant particulars, such as the condition of the skin, or some slight change in the diet or mode of life of the patient.

In laying so much stress upon mercurials, I do not wish you to understand that I am in the habit of administering these remedies carelessly, or with unnecessary frequency. On the contrary, I rarely prescribe them; nor would I use them in dysentery when mild purgatives will cure the disease readily; it is only in severe cases that I prefer the mercurial treatment, which is unquestionably the most effectual, and most rapid means of getting rid of the disorder. There is no necessity for producing decided ptyalism; a slight action upon the gums is sufficient to test the effects of the remedy.

I have explained to you the anatomical lesions at length, because your prognosis is, in severe cases, to a great degree, founded upon their extent, and you will perceive that a complete cure can only take place when the ulcerations of the intestine are healed.

